# Effects of Varying Levels of Fermented *Camellia oleifera* Seed Meal on Growth Performance, Biochemical Indices, and Intestinal Health in Nursery Pigs

**DOI:** 10.3390/ani16213432

**Published:** 2026-10-30

**Authors:** Baili Ren, Xiaobo Guo, Minfeng Ding, Xin Li, Jun Chen, Tiande Zou, Xingping Chen, Jinming You

**Affiliations:** 1 Jiangxi Province Key Laboratory of Animal Nutrition and Feed, Jiangxi Agricultural University, Nanchang 330045, China; rbaili@163.com (B.R.); guoxiaobo93@126.com (X.G.); dingminfeng0911@163.com (M.D.); 12017019@zju.edu.cn (X.L.); junchen@jxau.edu.cn (J.C.); tiandezou@jxau.edu.cn (T.Z.); cxp0315@jxau.edu.cn (X.C.); 2 College of Animal Science and Technology, Jiangxi Agricultural University, Nanchang 330045, China; 3 Ganzhou Animal Husbandry and Fisheries Research Institute, Ganzhou 341000, China

**Keywords:** *Camellia oleifera* seed meal, solid-state fermentation, nursery pigs, intestinal health

## Abstract

*Camellia oleifera* seed meal (CSM) is a protein-rich by-product from camellia oil production, but its utilization in animal diets is restricted by antinutritional compounds, especially tea saponins. This study evaluated the effects of solid-state fermented CSM, produced using *Bacillus subtilis*, *Aspergillus niger*, and *Trichoderma longibrachiatum*, on growth performance, antioxidant capacity, immune status, and intestinal health in weaned piglets, and identified the optimal dietary inclusion level. A total of 84 piglets (15.46 ± 0.10 kg) were randomly allocated to four treatments: a basal diet (control) or the basal diet supplemented with 2.5%, 5.0%, or 7.5% fermented CSM for 48 d. Compared with the control, 2.5% fermented CSM maintained normal growth performance while improving neutral detergent fiber digestibility, antioxidant activity, immune-related indices, jejunal morphology, and expression of tight junction proteins and MUC-2. The relative abundance of butyrate-producing *Collinsella* was altered, and colonic butyric acid showed a significant quadratic response to graded FCSM supplementation; meanwhile, isocaproic acid concentration was reduced. In contrast, supplementation at 5.0% or above negatively affected growth, nutrient utilization, and intestinal health. Overall, piglets exhibited an inverted U-shaped response to fermented CSM supplementation, with 2.5% identified as the most suitable inclusion level. This study provides a theoretical basis for the application of fermented CSM as a potential partial replacer of conventional feed ingredients in nursery pig diets.

## Simple Summary

The global swine industry is facing an increasing shortage of cost-effective protein feed resources. Camellia seed meal, a common by-product generated during camellia oil production, contains abundant protein but also carries digestion-impairing substances that restrict its direct use in pig feed. In this study, we treated camellia seed meal through fermentation with three beneficial microorganisms and evaluated the effects of different dietary addition levels on growth performance and intestinal health in weaned piglets. The results demonstrated that a 2.5% inclusion of fermented camellia seed meal maintained normal growth while improving nutrient digestibility, antioxidant capacity, immune function, and gut health in piglets. In contrast, addition levels of 5% or above suppressed growth and impaired intestinal health. These findings suggest that appropriately dosed fermented camellia seed meal can be used as a safe and economical alternative feed ingredient, providing a practical way to reuse agricultural by-products and reduce feeding costs in pig production.

## 1. Introduction

The global swine industry is currently facing a critical bottleneck characterized by a shortage in protein feed supply. As the conventional primary protein source, the application of soybean meal (SBM) is constrained by multiple factors, making it a key factor affecting the sustainable development of the swine industry [1]. Amidst the intensifying fuel–feed–food competition, seeking high-quality and low-cost feed raw materials is the core measure to promote the long-term sustainable development of the livestock industry [2]. As the world’s largest soybean importer, China relies on imports for over 85% of its soybean consumption, and the sources are highly concentrated. This makes the swine industry face multiple uncertainties, including geopolitical, trade, and climatic risks. SBM accounts for 15% of the total feed ingredients for livestock and poultry in China [3], and its supply stability directly affects feed costs and the equilibrium of meat supply and demand. Therefore, developing cost-effective and stably supplied alternative feed materials to SBM has become an imperative for the livestock and feed industries [4].

*Camellia oleifera* seed meal (CSM) is a by-product derived from oil extraction, which is abundant in protein, polyphenols, tea saponins, polysaccharides, and other nutritional and bioactive components, exhibiting great potential as a novel feed ingredient [5]. Tea saponins possess excellent surfactant characteristics and exhibit diverse biological activities, such as antioxidant, antibacterial, and anti-inflammatory effects [6,7]. Nevertheless, CSM is currently used primarily for tea saponin recovery and in the chemical and cosmetic industries [8]. A large proportion is discarded or only utilized in low-value scenarios [9], and its feed potential has not been fully exploited [10]. The main limiting factor is that CSM contains anti-nutritional factors (ANFs) such as 2~7% tannins, 1.5~3% alkaloids, and high levels of tea saponins [4]. The bitterness and toxicity of tea saponins are the key bottlenecks for their utilization in feed, and excessive ingestion can induce erythrocyte hemolysis and even mortality in animals [11]. Overcoming the limitations imposed by tea saponins has become the core technical challenge in the feed utilization of CSM.

As a green and efficient bioprocessing strategy, microbial fermentation can decrease antinutritional factors in CSM while generating beneficial metabolites such as peptides, organic acids, and phenolic compounds, resulting in improved nutritional quality and functional performance of the feed [12,13]. Previous studies have shown that Bacillus subtilis fermentation can enrich small-molecule active peptides and exhibit antioxidant activity [14]; solid-state fermentation (SSF) using *Lactobacillus plantarum* combined with cellulase can reduce the content of ANFs and increase crude protein and acid-soluble protein [15]; *Streptococcus*, *Bacillus*, and *Aspergillus niger* can synthesize saponin-degrading enzymes to convert tea saponins into less toxic derivatives [11]; moreover, co-fermentation with *Bacillus subtilis* and *Aspergillus niger* can achieve a degradation rate of 67.84% for tea saponins [16]. The above studies confirm that composite microbial fermentation is an effective approach to enhancing the safety of utilizing CSM in animal feed.

*Bacillus subtilis*, *Aspergillus niger*, and *Trichoderma longibrachiatum* are widely recognized as safe microorganisms for feed applications (GRAS) [17,18,19]. Previous studies have demonstrated that these strains can enhance growth performance, nutrient utilization, antioxidant status, and intestinal barrier integrity in pigs [20,21,22]. Nevertheless, no published studies have evaluated the application of CSM co-fermented with these three microorganisms in nursery pigs. Consequently, its effects on growth performance and intestinal health, as well as the underlying mechanisms, remain largely unknown, and the optimal dietary supplementation level has yet to be established. Therefore, we proposed that solid-state fermentation of CSM using a mixed culture of *Bacillus subtilis*, *Aspergillus niger*, and *Trichoderma longibrachiatum* could effectively reduce antinutritional factors and improve its nutritional value as a feed ingredient. The objective of this study was to evaluate the effects of dietary supplementation with different levels of fermented CSM on growth performance, antioxidant status, immune response, and intestinal health in nursery pigs, identify the most suitable inclusion level, explore the underlying mechanisms involved, and provide scientific evidence supporting the application of fermented CSM as a novel feed ingredient for nursery pigs.

## 2. Materials and Methods

### 2.1. Animal Ethics Statement

The animal experiment received ethical approval from the Animal Care and Use Committee of Jiangxi Agricultural University (Approval No. JXAULL-2025-06-12). All procedures were conducted in accordance with established regulations and ethical standards for animal research. The feeding trial took place during the period from June to August 2025.

### 2.2. Preparation of Fermented CSM

*Bacillus subtilis*, *Trichoderma longibrachiatum*, and *Aspergillus niger* were provided by Guangdong Microbial Culture Collection Center (GDMCC). *Bacillus subtilis* was activated in LB broth; *Trichoderma longibrachiatum* and *Aspergillus niger* were cultured on PDA medium at 30 °C for 4 days. Subsequently, the spores were harvested with sterile water and counted with a hemocytometer to prepare working microbial suspensions with a concentration of 1 × 10^7^ CFU/mL, respectively. The fermentation substrate consisted of CSM and wheat bran mixed at a ratio of 8:2 (*w*/*w*), which was then sieved through a 40-mesh screen, with 1% (*w*/*w*) molasses added as a supplemental carbon source. The composite microbial suspension (with a volume ratio of 1:1:1 for the three working suspensions) was inoculated at a dose of 24% (*v*/*w*), and the substrate-to-water ratio (substrate: distilled water) was adjusted to 1:0.7 (*w*/*v*). After being thoroughly mixed, the fermentation substrate was transferred to a fermenter and subjected to solid-state fermentation at 33 °C for 4 d, with daily manual agitation. Upon completion of the fermentation process, the product was dried at 60 °C for 48 h, ground to pass a 40-mesh sieve, and stored at 4 °C in sealed containers. Tea saponin concentrations were determined using the vanillin–sulfuric acid colorimetric method. Raw *Camellia oleifera* seed meal (CSM) contained 203.49 ± 8.40 mg/g dry matter tea saponins. Following solid-state fermentation with the mixed microbial inoculant, residual tea saponin content fell to 26.94 ± 1.52 mg/g dry matter, corresponding to an 86.76% degradation rate. This degradation performance is consistent with previous studies using the same strain combination and similar solid-state fermentation process for CSM, which also reported effective tea saponin decomposition and general improvement of nutritional components via microbial metabolism [11,16]. A complete schematic diagram of the solid-state fermentation process of *Camellia oleifera* seed meal is provided in Supplementary Figure S1.

### 2.3. Experimental Diets

The basal diet, based on corn and soybean meal, was formulated to satisfy the nutritional requirements of 15–30 kg growing pigs according to NRC [23] recommendations. Piglets were assigned to four dietary treatments: a basal diet control and three diets containing 2.5%, 5.0%, or 7.5% fermented CSM. In the supplemented diets, fermented CSM was incorporated by substituting equal proportions of extruded corn and soybean meal. The diets were formulated to simulate the practical application scenario in commercial production, in which fermented CSM directly replaced extruded corn and soybean meal in equal proportion without additional energy or amino acid calibration. Detailed dietary ingredients and nutrient compositions, including the calculated levels of neutral detergent fiber (NDF) and acid detergent fiber (ADF) as the dietary baseline for fiber utilization interpretation, are provided in Table 1.

### 2.4. Animals and Management

Eighty-four healthy Duroc × Landrace × Yorkshire (DLY) nursery pigs (50 d of age) with an initial body weight of 15.46 ± 0.10 kg (mean ± SD) were selected for the feeding trial. Animals were randomly distributed into four dietary treatments according to their initial BW, with seven replicate pens per treatment and three pigs housed in each pen. Pens measured 2.0 m × 1.8 m × 0.9 m and were equipped with a feeder and a stainless-steel nipple drinker. Throughout the experimental period, pigs had free access to feed and drinking water. The feeding trial was carried out in a climate-controlled, fully enclosed nursery barn. Environmental conditions were maintained at 24–26 °C with a relative humidity of 55–65%, using both natural ventilation and negative-pressure mechanical ventilation. The experiment extended over 48 d and comprised three phases: an adaptation period (d 1–7), a growth performance assessment period (d 8–48), and a metabolism trial period (d 46–48). All pigs received the basal diet during the 7-d adaptation period. Thereafter, the corresponding experimental diets were provided throughout the growth performance trial. For the metabolism trial, the daily feed allocation of each pen was adjusted to match the average daily feed intake (ADFI) determined during the preceding growth performance period.

### 2.5. Determination of Growth Performance

The pigs were weighed after fasting in the morning on days 1 and 48 of the growth performance trial period, respectively, to record their body weight (BW). Feed was offered daily at 08:00, 14:00, and 20:00, with the amount of feed offered and refusals recorded on a pen basis. The average daily gain (ADG), average daily feed intake (ADFI), and feed-to-gain ratio (F:G) were calculated.

### 2.6. Sample Collection and Processing

During the 3-d metabolism trial, fresh fecal samples were collected daily from each pen, transferred into sealed plastic bags, and stored at −20 °C until analysis. At the conclusion of the experiment, one pig whose body weight was closest to the pen mean was selected from each replicate for sampling. Blood was obtained by anterior vena cava puncture and kept at room temperature for 30 min before centrifugation at 3000× *g* for 15 min. The resulting serum was harvested and stored at −20 °C.

Following blood collection, pigs were anesthetized and humanely euthanized. The middle segments of the duodenum, jejunum, and ileum were excised immediately. Intestinal samples were divided into two portions: one was fixed in 4% paraformaldehyde for histological analysis, whereas the mucosa from the other portion was carefully scraped, rapidly frozen in liquid nitrogen, and preserved at −80 °C. In addition, colonic digesta from the mid-colon was aseptically collected, snap-frozen in liquid nitrogen, and stored at −80 °C for subsequent analysis.

### 2.7. Determination of Apparent Nutrient Digestibility 

Feed and fecal samples were analyzed for crude protein (CP), ether extract (EE), ash, neutral detergent fiber (NDF), acid detergent fiber (ADF), calcium, and total phosphorus following the procedures specified in the National Standards of the People’s Republic of China (GB/T 6432-2018 [24], GB/T 6433-2006 [25], GB/T 6438-2007 [26], GB/T 20806-2006 [27], NY/T 1459-2007 [28], GB/T 6436-2018 [29], GB/T 6437-2018 [30], and GB/T 23742-2009 [31]). Acid-insoluble ash (AIA) served as an internal indicator for determining apparent nutrient digestibility.

### 2.8. Determination of Serum and Intestinal Mucosal Indices

Serum and jejunal mucosal concentrations of IgA, IgG, IgM, TNF-α, IFN-γ, IL-1, IL-6, and IL-10 were determined using ELISA kits (Shanghai Enzyme-linked Biotechnology Co., Ltd., Shanghai, China). The activities of T-AOC, T-SOD, GSH-Px, and CAT, together with MDA content, were measured using commercial assay kits purchased from Nanjing Jiancheng Bioengineering Institute (Nanjing, China). Jejunal mucosal homogenates were prepared by homogenizing 0.10–0.15 g of tissue with nine volumes (*w*/*v*) of chilled saline in an ice bath. Following centrifugation, the supernatant was collected for subsequent analyses according to the kit instructions. All assays were conducted in strict accordance with the manufacturers’ protocols.

### 2.9. Intestinal Morphological Analysis

The duodenal, jejunal, and ileal tissues were fixed in 4% paraformaldehyde (Servicebio Technology Co., Ltd., Wuhan, China), dehydrated using a graded ethanol series prepared from anhydrous ethanol (Servicebio Technology Co., Ltd., Wuhan, China), cleared with xylene (Servicebio Technology Co., Ltd., Wuhan, China), and subsequently embedded in paraffin (Servicebio Technology Co., Ltd., Wuhan, China). Paraffin blocks were sectioned into 2-µm slices, followed by hematoxylin and eosin (H&E) staining (Servicebio Technology Co., Ltd., Wuhan, China) and mounting.. Histological observations were performed using a DM300 microscope at 10× magnification (Leica Microsystems, Wetzlar, Germany). For each sample, 10 intact villi were randomly selected and analyzed with Saiviewer v3.0 software (Shenzhen Saiviewer Technology Co., Ltd., Shenzhen, China) to determine villus height (VH; measured from the villus tip to the villus–crypt junction) and crypt depth (CD; measured from the crypt base to the villus–crypt junction), and the VH:CD ratio was subsequently calculated. Ten well-oriented villus–crypt structures from each intestinal segment of every pig were evaluated, and the mean values were used for statistical analysis.

### 2.10. RNA Extraction and Quantitative Real-Time PCR (RT-qPCR)

Jejunal mucosal total RNA was extracted using an RNA isolation kit (Accurate Biology, Changsha, China). The quality and concentration of RNA were measured using a DU800 spectrophotometer (Beckman Coulter, Brea, CA, USA), followed by cDNA synthesis with a reverse transcription kit (Accurate Biology, Changsha, China). Quantitative real-time PCR was conducted using SYBR Green I chemistry, and β-actin was used as the housekeeping gene. Relative mRNA abundance of target genes was quantified using the 2^−ΔΔCt^ method. Detailed primer sequences for all detected target and reference genes are listed in Supplementary Table S1.

### 2.11. High-Throughput Sequencing of the Colonic Microbiota 16S rRNA Gene

Microbial genomic DNA was extracted from colonic digesta samples using the E.Z.N.A.^®^ Soil DNA Kit (Omega Bio-tek Inc., Norcross, GA, USA). DNA quality and concentration were evaluated by 1% agarose gel electrophoresis and a NanoDrop 2000 spectrophotometer (Thermo Fisher Scientific, Waltham, MA, USA). Full-length 16S rRNA genes were amplified by PCR for 27 cycles using the barcoded universal primers 27F (5′-AGRGTTYGATYMTGGCTCAG-3′) and 1492R (5′-RGYTACCTTGTTACGACTT-3′). The resulting amplicons were purified with magnetic beads and quantified using a Qubit 4 fluorometer (Thermo Fisher Scientific, Waltham, MA, USA) prior to library construction (Thermo Fisher Scientific, Waltham, MA, USA). Subsequently, SMRTbell libraries were prepared and sequenced on the PacBio Sequel IIe platform (Shanghai Majorbio Bio-pharm Technology Co., Ltd., Shanghai, China). Raw sequencing data were processed with SMRT-Link v11.0 software to obtain high-fidelity (HiFi) reads for downstream analysis. These reads were denoised using the DADA2 plugin in QIIME 2 (v2023.5) to obtain amplicon sequence variants (ASVs). After removing chloroplast and mitochondrial contamination, the sequences were rarefied to 6000 reads per sample (yielding an average coverage of 99.12%). Taxonomic annotation was performed based on the Silva v138.2 database. Alpha diversity indices were calculated, and differences in community structure were analyzed via principal coordinate analysis (PCoA) based on Bray–Curtis distances coupled with analysis of similarities (ANOSIM). Linear discriminant analysis effect size (LEfSe) (LDA > 2.0, *p* < 0.05) was employed to identify differentially abundant taxa. All bioinformatics analyses were conducted on the Majorbio Cloud Platform.

### 2.12. Determination of Short-Chain Fatty Acids in Colonic Contents

Short-chain fatty acids (SCFAs), including acetic, propionic, isobutyric, butyric, isovaleric, valeric, caproic, and isocaproic acids, were quantified in colonic contents using an Agilent 8890B-7000D GC–MS system equipped with an HP-FFAP capillary column (30 m × 0.25 mm × 0.25 μm) (Agilent Technologies, Santa Clara, CA, USA). 2-Ethylbutyric acid (Sigma-Aldrich, St. Louis, MO, USA) served as the internal standard.. For sample preparation, 25 mg of colonic digesta was suspended in 0.5% phosphoric acid solution, extracted with n-butanol, and then analyzed by GC–MS. The GC temperature program consisted of an initial temperature of 80 °C, increasing to 120 °C at 20 °C/min, then to 160 °C at 5 °C/min, and finally to 220 °C at 20 °C/min with a 3-min hold. The mass spectrometer was operated in selected ion monitoring (SIM) mode with an electron ionization (EI) source set at 70 eV. Metabolite concentrations were quantified using standard calibration curves generated in MassHunter v10.0. Quality control was assessed by inserting one QC sample after every 10 samples, with peak area RSD values maintained below 15%. Data analysis was conducted through the Majorbio Cloud Platform.

### 2.13. Statistical Analysis

Statistical analyses were performed using IBM SPSS Statistics 25.0 (IBM Corp., Armonk, NY, USA). Data were analyzed by one-way ANOVA after verification of variance homogeneity using Levene’s test. Treatment means were compared using Duncan’s multiple range test. Linear and quadratic effects of increasing FCSM supplementation levels were assessed by orthogonal polynomial contrasts. Data are presented as mean ± SEM. Differences were considered significant at *p* < 0.05 and highly significant at *p* < 0.01, whereas 0.05 ≤ *p* < 0.10 was regarded as a tendency toward significance.

## 3. Results

### 3.1. Growth Performance and Apparent Nutrient Digestibility

As shown in Table 2, dietary supplementation of fermented CSM highly significantly affected the final body weight (FBW), average daily gain (ADG), average daily feed intake (ADFI), and feed-to-gain ratio (F:G) of nursery pigs (*p* < 0.001). Compared with the CON group, no significant differences were observed for the above indices in the FCSM2.5 group (*p* > 0.05); however, the FBW, ADG, and ADFI in the FCSM5 and FCSM7.5 groups were significantly decreased, and the F:G was significantly increased (*p* < 0.05). As the inclusion level of FCSM increased, the FBW, ADG, and ADFI decreased linearly and quadratically (*p* ≤ 0.001), whereas the F:G increased linearly (*p* < 0.001) and quadratically (*p* = 0.029).

With respect to nutrient utilization, dietary supplementation with fermented CSM significantly influenced the apparent digestibility of crude protein (CP) and neutral detergent fiber (NDF) (*p* = 0.036 and *p* < 0.001, respectively). The FCSM2.5 group showed greater CP digestibility than the FCSM7.5 group (*p* < 0.05). As the dietary inclusion level of fermented CSM increased, CP digestibility displayed a linear decline (*p* = 0.050) together with a highly significant quadratic response (*p* < 0.001). Similarly, the apparent digestibility of ADF was markedly higher in the FCSM2.5 group than in the CON and FCSM7.5 groups (*p* < 0.01), and a significant quadratic effect was also detected (*p* < 0.001). In contrast, no treatment-related differences were observed for the digestibility of other nutrients (*p* > 0.05).

### 3.2. Effects of Fermented Camellia oleifera Seed Meal on Antioxidant Capacity in the Jejunum and Serum of Nursery Pigs

As presented in Table 3, dietary supplementation with FCSM markedly influenced antioxidant-related indices in both the jejunal mucosa and serum. In the jejunal mucosa, all FCSM-treated groups showed higher T-AOC values than the CON group (*p* < 0.05), with a highly significant linear response to increasing FCSM inclusion levels (*p* < 0.001). Compared with the CON group, T-SOD activity was significantly elevated in all supplemented groups (*p* < 0.01), and a significant quadratic effect was detected (*p* = 0.002). In addition, GSH-Px activity increased linearly with increasing dietary FCSM levels (*p* = 0.033). However, CAT activity and MDA concentration were not affected by dietary treatment (*p* > 0.05). 

In serum, pigs receiving FCSM supplementation exhibited significantly greater T-AOC and T-SOD activities than those in the CON group (*p* < 0.05). The T-AOC showed a significant linear increase (*p* = 0.002) and a tendency toward a quadratic response (*p* = 0.067) as the supplementation level increased. Likewise, T-SOD activity was affected by both linear and quadratic responses (*p* < 0.001). No significant treatment effects were observed for serum GSH-Px activity, CAT activity, or MDA content (*p* > 0.05).

### 3.3. Effects of Fermented Camellia oleifera Seed Meal on Jejunal and Serum Immune Functions in Nursery Pigs

As presented in Table 4, dietary FCSM supplementation altered several immune-related parameters in both the jejunal mucosa and serum. In the jejunal mucosa, pigs in the FCSM2.5 group exhibited higher concentrations of IL-10 and IgA than those in the CON and FCSM7.5 groups (*p* < 0.05). In contrast, IFN-γ levels were greater in the FCSM7.5 group than in the CON and FCSM2.5 groups (*p* < 0.05). Increasing dietary FCSM levels resulted in significant quadratic responses for IL-10 and IgA (*p* ≤ 0.009), whereas IFN-γ showed a highly significant linear increase (*p* < 0.001). No significant treatment effects were detected for IL-6, TNF-α, IgG, or IgM (*p* > 0.05).

In serum, IgA concentration was significantly elevated in the FCSM2.5 group compared with the CON group (*p* < 0.05). Conversely, serum IL-10 concentration was lower in the FCSM7.5 group than in the other treatment groups (*p* < 0.05). The FCSM7.5 group also exhibited reduced IgG levels relative to the CON and FCSM2.5 groups (*p* < 0.05), while no difference was observed compared with the FCSM5 group (*p* > 0.05). Both IL-10 and IgG showed significant linear declines with increasing FCSM inclusion levels (*p* ≤ 0.023), whereas IgA displayed a significant quadratic response (*p* = 0.021). The remaining serum immune parameters were not affected by dietary treatment (*p* > 0.05). To further verify the correlation between serum and jejunal mucosal immune status, Pearson correlation analysis was performed on key immune indicators. The results showed that serum IgA was extremely significantly positively correlated with jejunal IgA (r = 0.74, *p* < 0.01); serum IL-10 was extremely significantly positively correlated with jejunal IL-10 (r = 0.64, *p* < 0.01); serum IFN-γ showed a weak positive correlation with jejunal IFN-γ, but without statistical significance (r = 0.29, *p* > 0.05). Overall, serum immune indicators can reliably reflect the local immune status of the jejunal mucosa, with better consistency observed for humoral immune indicators and anti-inflammatory factors.

### 3.4. Effects of Fermented Camellia oleifera Seed Meal on Intestinal Morphology in Nursery Pigs (H&E Staining, 100×)

As shown in Table 5, dietary inclusion of fermented CSM did not affect villus height (VH), crypt depth (CD), or the VH ratio in either the duodenum or ileum (*p* > 0.05). In contrast, significant differences were observed in jejunal VH and VH ratio among treatments (*p* ≤ 0.032). Specifically, pigs in the FCSM2.5 group exhibited greater jejunal VH than those in the CON and FCSM7.5 groups (*p* < 0.05). Although the VH value in the FCSM7.5 group was lower than that in the FCSM2.5 group (*p* < 0.05), it did not differ significantly from the CON group. Similarly, the VH ratio in the jejunum was significantly elevated in the FCSM2.5 group compared with the CON and FCSM7.5 groups (*p* < 0.05). Both jejunal VH and VH ratio displayed significant quadratic responses to increasing dietary FCSM levels (*p* < 0.01).

**Figure 1:**
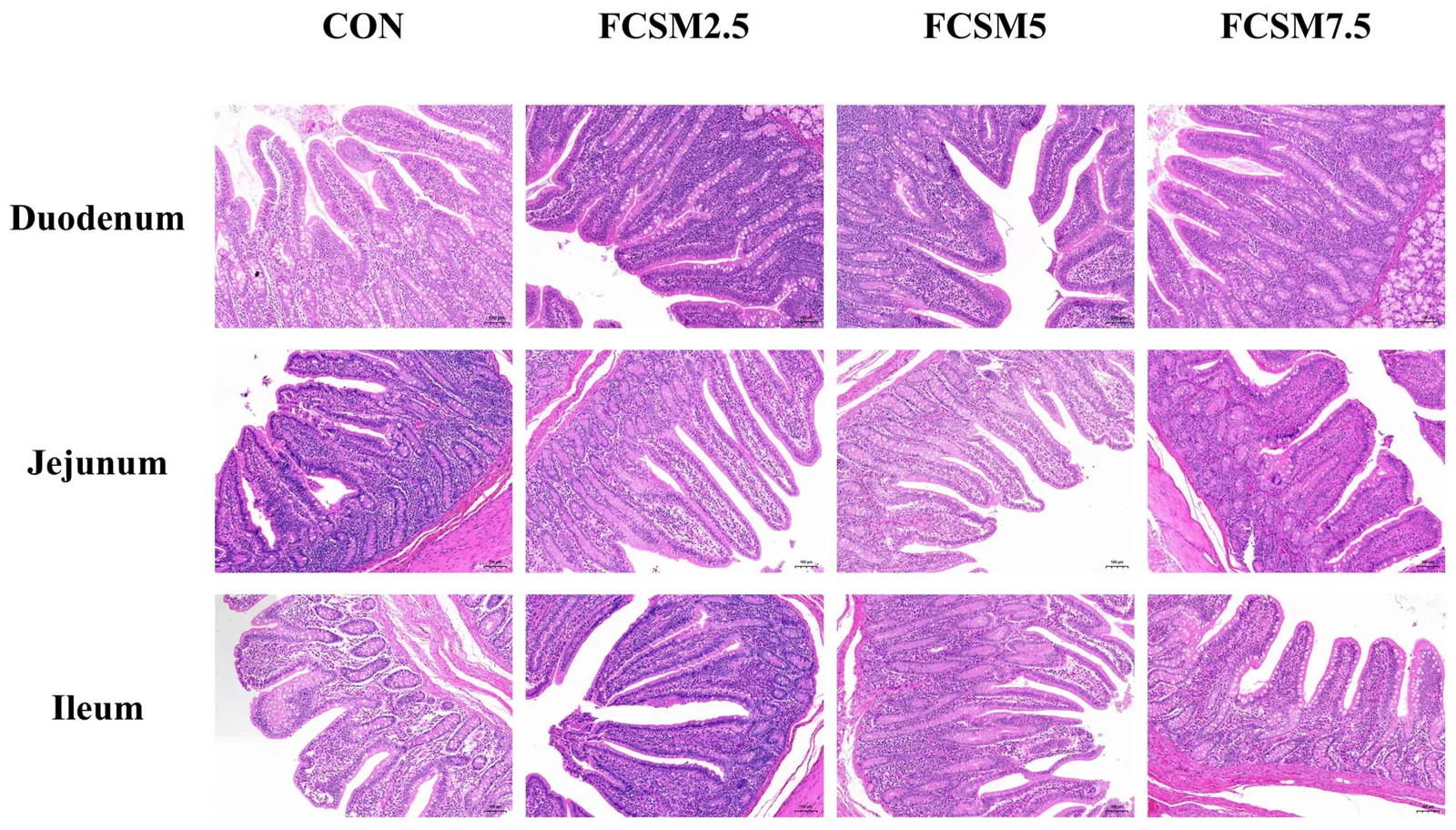
Effects of Fermented *Camellia oleifera* Seed Meal on Intestinal Morphology in Nursery Pigs (H&E Staining, 100×; Scale Bar = 100 μm).

The histological observations shown in Figure 1 were in agreement with the morphometric data. Jejunal villi from pigs fed the 2.5% FCSM diet were structurally intact and arranged in a compact and orderly manner. In contrast, villus atrophy, fragmentation, and epithelial cell shedding were evident in the FCSM7.5 group.

### 3.5. Effects of Fermented Camellia oleifera Seed Meal on the Relative mRNA Expression of Related Genes in the Jejunal Mucosa of Nursery Pigs

**Figure 2:**
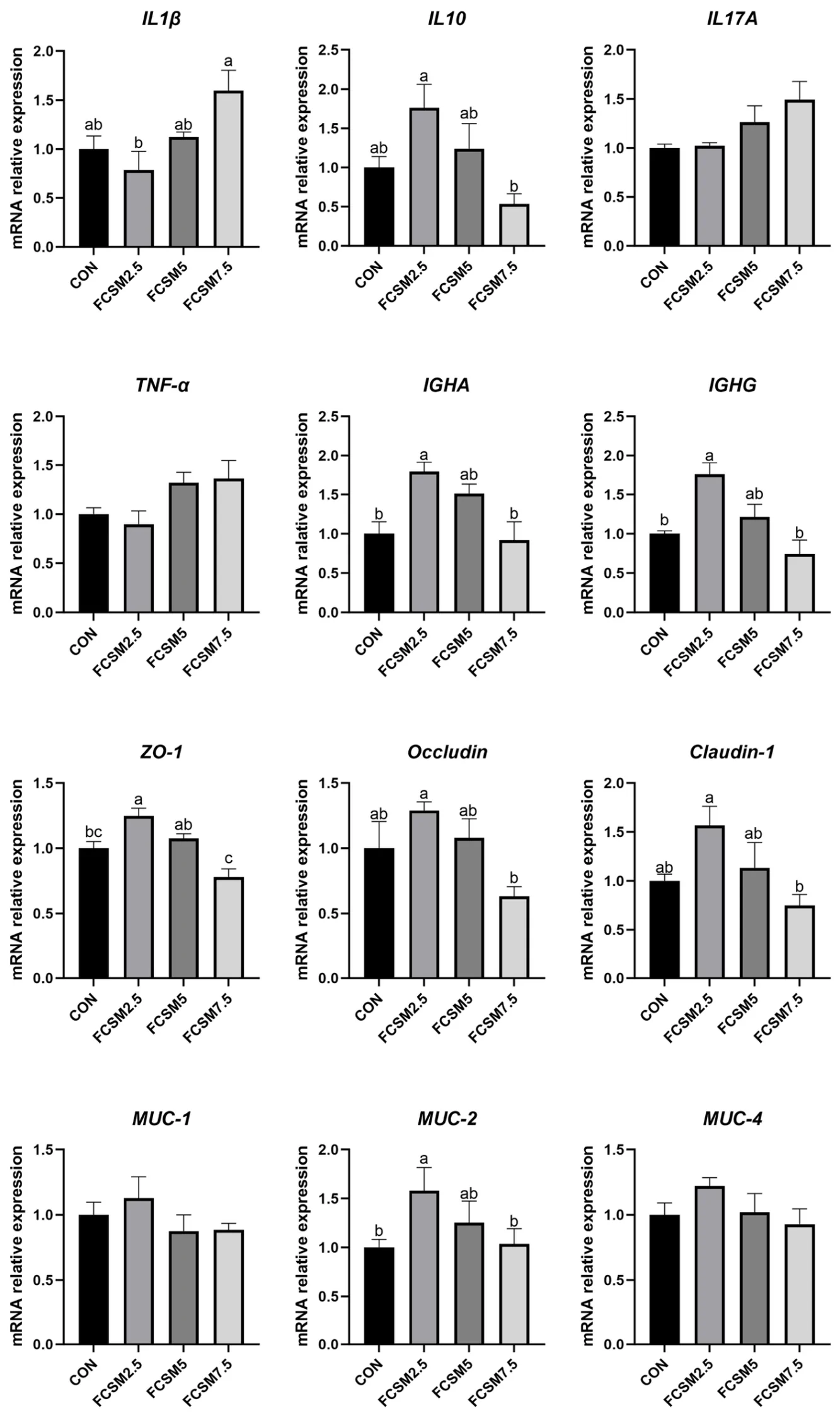
Effects of Fermented *Camellia oleifera* Seed Meal on the Relative mRNA Expression of Barrier and Immune-related Genes in the Jejunal Mucosa of Nursery Pigs. Bars with different superscript lowercase letters differ significantly (*p* < 0.05), whereas those with the same or no letter do not differ (*p* > 0.05). CON, basal diet; FCSM2.5, FCSM5, and FCSM7.5, basal diet supplemented with 2.5%, 5.0%, and 7.5% fermented *Camellia oleifera* seed meal, respectively. SEM, standard error of the mean. *n* = 7 per treatment.

As illustrated in Figure 2, dietary supplementation with fermented CSM significantly influenced the jejunal mRNA expression of immune- and inflammation-related genes, including IGHA, IGHG, IL1B, and IL10 (*p* < 0.05). In contrast, no significant treatment effects were observed for IL17A or TNF-α expression (*p* > 0.05). Compared with the CON group, pigs fed the 2.5% fermented CSM diet exhibited significantly greater expression levels of IGHA and IGHG (*p* < 0.05). Moreover, the FCSM7.5 group showed a higher expression level of the pro-inflammatory gene IL1B than the FCSM2.5 group (*p* < 0.05). Conversely, the expression of the anti-inflammatory gene IL10, together with the immunoglobulin-related genes IGHA and IGHG, was significantly reduced in the FCSM7.5 group relative to the FCSM2.5 group (*p* < 0.05). No significant differences were detected between the FCSM7.5 and CON groups for any of the above inflammation- or immunity-associated genes (*p* > 0.05).

In addition to immune- and inflammation-related genes, fermented CSM also affected the expression of genes associated with intestinal barrier integrity. Significant treatment effects were detected for ZO-1, Occludin, Claudin-1, and MUC2 expression in the jejunal mucosa (*p* < 0.05), whereas MUC1 and MUC4 expression remained unchanged (*p* > 0.05). Relative to the CON group, the FCSM2.5 group showed significantly increased expression of ZO-1 and MUC2 (*p* < 0.05). Moreover, the expression levels of ZO-1, Occludin, and Claudin-1 were significantly reduced in the FCSM7.5 group compared with the FCSM2.5 group (*p* < 0.05).

### 3.6. Effects of Fermented Camellia oleifera Seed Meal on the Colonic Microbiota in Nursery Pigs

**Figure 3:**
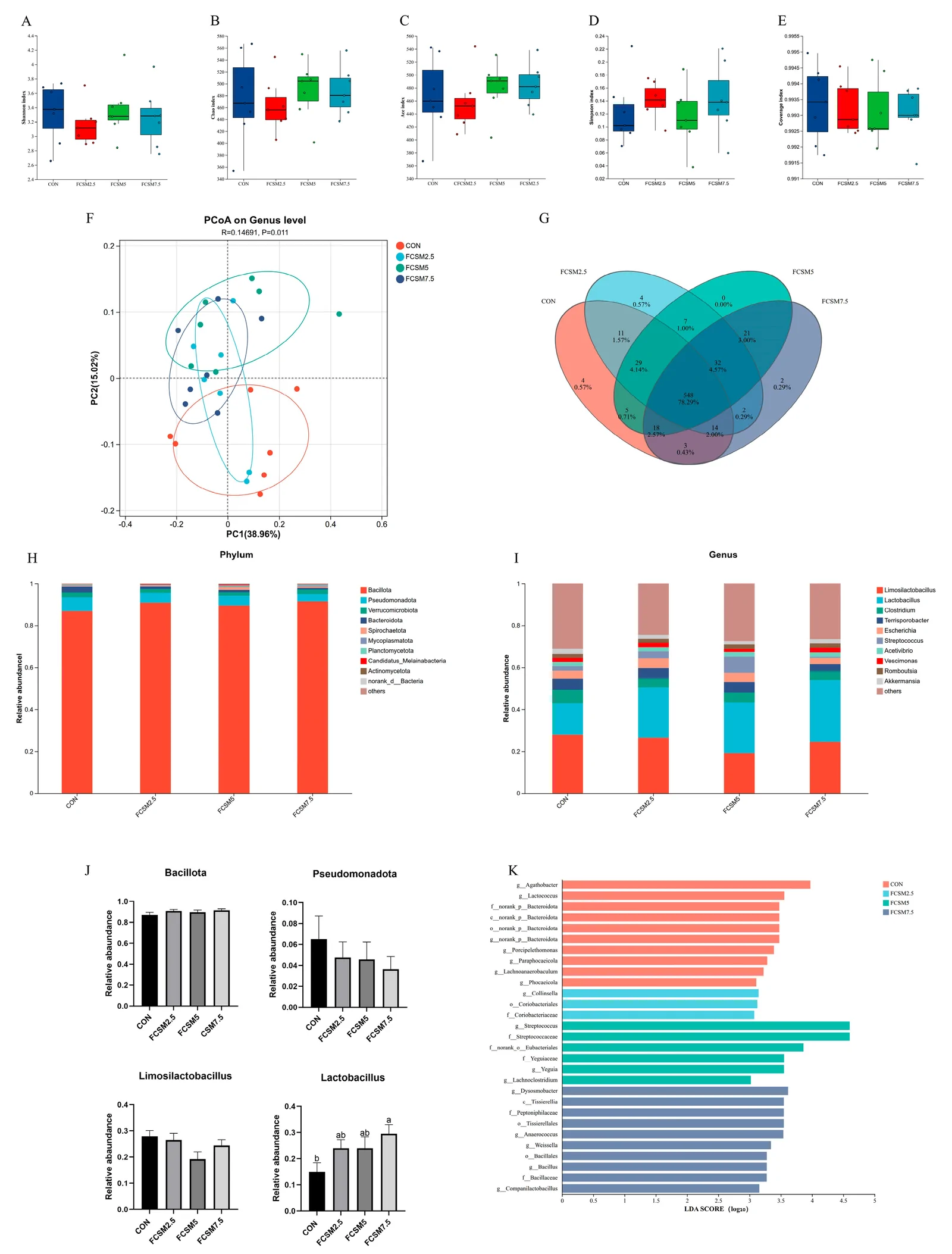
Effects of fermented *Camellia oleifera* seed meal on colonic microbiota in nursery pigs. (**A**–**E**) Box plots of alpha diversity indices, including Shannon index (**A**), Chao1 richness index (**B**), Ace richness index (**C**), Simpson diversity index (**D**), and Good’s coverage (**E**). (**F**) Principal coordinate analysis (PCoA) based on Bray–Curtis distances at the genus level; ellipses represent 95% confidence intervals for each group, and ANOSIM confirmed significant differences in microbial community structure among treatments (R = 0.14691, *p* = 0.011). (**G**) Venn diagram showing the number of shared and unique operational taxonomic units (OTUs) across the four treatment groups; numbers inside the regions indicate OTU counts, and percentages represent their proportion of the total OTUs. (**H**,**I**) Stacked bar charts showing the relative abundance of dominant bacterial phyla (**H**) and genera (**I**) in each treatment group. (**J**) Comparison of the relative abundance of two dominant phyla (*Bacillota* and *Pseudomonadota*) and two dominant genera *(Limosilactobacillus* and *Lactobacillus*) among groups; different lowercase letters above bars indicate significant differences (*p* < 0.05). (**K**) Linear discriminant analysis effect size (LEfSe) analysis identifying differentially abundant taxa among treatments; bars with different colors represent taxa significantly enriched in the corresponding group, and bar length indicates the LDA score (threshold: LDA > 2.0, *p* < 0.05). CON = basal diet; FCSM2.5, FCSM5, FCSM7.5 = basal diet supplemented with 2.5%, 5.0%, and 7.5% fermented *C. oleifera* seed meal, respectively.

As illustrated in Figure 3, Good’s coverage values (reflecting the proportion of microbial sequences captured by sequencing; a value closer to 1 indicates sufficient sequencing depth to represent the real community structure) exceeded 0.99 for all samples, indicating adequate sequencing depth. Analysis of α-diversity showed that dietary FCSM supplementation did not significantly affect the Shannon, Chao1, Ace, or Simpson indices (*p* > 0.05), although lower Shannon, Chao1, and Ace values were observed in the FCSM2.5 group. At the genus level, principal coordinate analysis (PCoA) based on Bray–Curtis distances combined with ANOSIM revealed statistically significant but weak differences in colonic microbial community structure across groups (R = 0.14691, *p* = 0.011). The OTU Venn diagram identified 700 nonredundant OTUs across all groups, of which 548 were shared by all treatments, representing 78.29% of the total OTUs. Taxonomic composition analysis demonstrated that *Bacillota* and *Pseudomonadota* were the dominant phyla, collectively accounting for more than 90% of the microbial community. At the genus level, *Limosilactobacillus* and *Lactobacillus* were the most abundant genera, together comprising over 50% of the total relative abundance. LEfSe analysis (LDA score > 2.0) further identified group-specific bacterial biomarkers. *Agathobacter* and *Lactococcus* were enriched in the CON group, *Collinsella* was characteristic of the FCSM2.5 group, and Streptococcus was enriched in the FCSM5 group, whereas *Lactobacillus*, *Weissella*, and *Bacillus* were predominantly associated with the FCSM7.5 group.

### 3.7. Effects of Fermented Camellia oleifera Seed Meal on Colonic Short-Chain Fatty Acids in Nursery Pigs

As presented in Table 6, dietary FCSM supplementation influenced the concentration of certain colonic short-chain fatty acids. Although no significant differences in butyric acid concentration were detected among treatments (*p* = 0.128), a significant quadratic response was observed with increasing FCSM inclusion levels (*p* = 0.025). In contrast, the concentrations of acetic, propionic, valeric, caproic, isobutyric, and isovaleric acids remained unaffected by dietary treatment (*p* > 0.05).

Among the branched-chain fatty acids, isocaproic acid was significantly affected by FCSM supplementation (*p* = 0.006) and exhibited a linear decrease as dietary inclusion levels increased (*p* = 0.002). Compared with the CON group, pigs in the FCSM2.5 and FCSM5 groups had lower isocaproic acid concentrations (*p* < 0.05), whereas the FCSM7.5 group did not differ significantly from the CON group (*p* > 0.05).

### 3.8. Correlation Analysis Between Key Differential Genera and Indicators of Intestinal Health and Growth Performance

**Figure 4:**
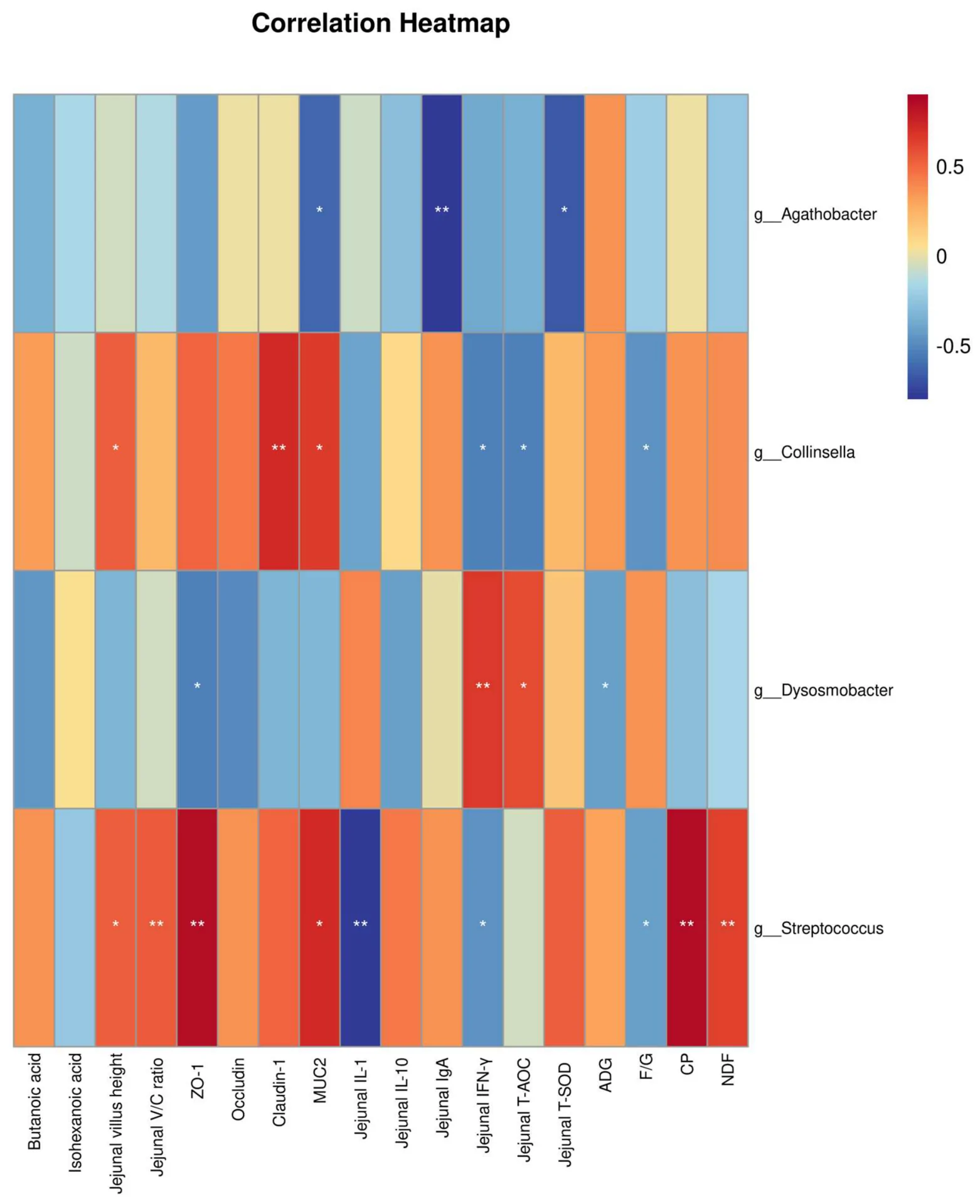
Correlations between key differential genera and indices of intestinal health and growth performance. Red and blue represent positive and negative correlations, respectively. * *p* < 0.05 and ** *p* < 0.01 indicate significant and highly significant correlations, respectively. ADG = average daily gain; F/G = feed-to-gain ratio; NDF = neutral detergent fiber digestibility; VH = jejunal villus height; V/C = villus height-to-crypt depth ratio; T-AOC = total antioxidant capacity; T-SOD = total superoxide dismutase; IL-1β = interleukin-1β; IL-10 = interleukin-10; IFN-γ = interferon-γ; IgA = immunoglobulin A.

As shown in Figure 4, significant associations were observed between the key differential bacterial genera and parameters related to intestinal health and growth performance in nursery pigs. *Collinsella*, which was enriched in the FCSM2.5 group, showed positive correlations with Claudin-1 expression, MUC2 expression, and jejunal villus height (*p* < 0.05), whereas negative correlations were detected with jejunal IFN-γ concentration and feed-to-gain ratio (*p* < 0.05). In addition, *Collinsella* tended to be positively associated with ZO-1 expression and average daily gain (*p* < 0.10). 

*Streptococcus*, the characteristic genus of the FCSM5 group, was positively correlated with several nutrient digestibility and intestinal health indicators, while exhibiting negative correlations with pro-inflammatory factors and feed-to-gain ratio (*p* < 0.05). *Agathobacter*, which was enriched in the CON group, was negatively associated with jejunal IgA, T-SOD activity, and MUC2 expression (*p* < 0.05). Furthermore, *Dysosmobacter*, enriched in the FCSM7.5 group, showed a highly significant positive correlation with the pro-inflammatory cytokine IFN-γ in the jejunum (*p* < 0.01) and was negatively correlated with ZO-1 expression and average daily gain (*p* < 0.05).

## 4. Discussion

As a potential substitute for soybean meal, *Camellia oleifera* seed meal (CSM) contains abundant protein, dietary fiber, and a variety of bioactive substances, making it a promising feed resource for mitigating soybean meal shortages and lowering production costs. Nevertheless, its practical utilization in animal production remains limited by anti-nutritional components, including tea saponins and tannins. In particular, the strong bitterness associated with tea saponins substantially reduces feed acceptability and restricts the wider application of CSM in livestock and poultry diets [32]. Therefore, developing effective processing strategies to reduce these limitations has become an important research objective. In the present study, the effects of different dietary levels of compound probiotic-fermented CSM on nursery pigs were comprehensively evaluated. The results demonstrated a distinct dose-dependent response, exhibiting an inverted U-shaped pattern in which moderate supplementation produced beneficial effects, whereas excessive inclusion exerted adverse impacts. Notably, supplementation with 2.5% fermented CSM (FCSM2.5) did not negatively affect final body weight, average daily gain, average daily feed intake, or feed-to-gain ratio, indicating that this inclusion level can be considered safe and effective under the conditions of the current experiment. This finding aligns with the general application patterns of fermented plant-based feeds in pig production; however, the key innovation of this study lies in explicitly identifying the safe dose window and toxicity threshold of fermented CSM in nursery pig diets for the first time. The underlying beneficial mechanism is that saponin-degrading enzymes synthesized by the compound probiotics (*Bacillus subtilis*, *Aspergillus niger*, and *Trichoderma longibrachiatum*) during fermentation can effectively cleave the tetrasaccharide unit structure linking the aglycone polar group and the β-1,4-glycosidic bond in tea saponins [33]. Given the lack of β-glucuronidase capable of cleaving this glycosidic bond in the porcine digestive tract, untreated CSM significantly hinders the digestive process. In contrast, fermentation converts tea saponins into derivatives with weaker hemolytic activity and lower toxicity, simultaneously improving feed palatability [11,34]. When the inclusion level reached 5.0% or higher, the growth performance of nursery pigs significantly decreased, exhibiting a highly significant linear deterioration. This fundamentally reflects an imbalance between the cumulative effects of residual ANFs and the beneficial effects: even after fermentation, high inclusion levels introduce residual tea saponins that exceed the tolerance threshold of nursery pigs. Their bitter and astringent taste reduces feed intake, while the ANFs directly stimulate the intestinal mucosa, compromise barrier integrity, and induce inflammation, ultimately triggering a vicious cycle of “decreased feed intake, impaired digestion and absorption, and hindered growth” [35]. Compared with previous reports, the present study not only corroborates the beneficial effects of fermented CSM on nursery pigs but also, for the first time, identifies an optimal dietary inclusion threshold, thereby filling the knowledge gap regarding its dose-dependent responses. In terms of nutrient utilization, pigs fed the FCSM2.5 diet exhibited greater apparent digestibility of neutral detergent fiber (NDF) than those in the CON group. This observation agrees with the findings of Yong et al. [36], suggesting that microbial fermentation may modify the physicochemical characteristics of dietary fiber and increase the residence time of digesta within the gastrointestinal tract. Consequently, a more favorable environment is created for the growth and activity of fibrolytic microorganisms, leading to enhanced fiber degradation and utilization. However, when the inclusion level exceeded 5%, the NDF digestibility significantly declined, which may be attributed to the inhibition of intestinal fibrolytic microbial proliferation by high doses of residual ANFs [37]. Furthermore, the crude protein digestibility in the FCSM7.5 group exhibited a decreasing trend, corroborating the findings of Farhana et al. [38] that once the total amount of residual saponins and tannins in plant-based ingredients exceeds a certain threshold, it continues to significantly inhibit protein digestion despite processing. The growth suppression at high inclusion levels can be attributed to three synergistic mechanisms underlying this dose-dependent effect: first, residual tea saponins exceed the tolerance threshold of nursery pigs, inducing intestinal inflammation and impairing nutrient digestibility [35]; second, excessive insoluble fiber accelerates intestinal transit, shortens nutrient retention time and dilutes dietary nutrient density, further reducing nutrient utilization efficiency; third, the bitter taste of residual saponins reduces feed palatability and daily feed intake, which in turn aggravates digestive impairment and growth inhibition in a vicious cycle. In summary, the significant quadratic effects of fermented CSM inclusion levels on the growth performance and nutrient digestibility of nursery pigs are essentially the result of a dynamic balance between beneficial effects (ANF degradation, nutritional value enhancement) and detrimental effects (residual toxicity, negative impacts of high fiber), which provides core data support for its precise application.

Antioxidant capacity and immune function are core indicators for evaluating the health status of nursery pigs [39]. As a crucial link connecting nutrient absorption, immunity, and disease resistance, intestinal health and its homeostasis directly impact overall health and growth performance; among these, oxidative stress and inflammatory responses are the primary threats compromising intestinal integrity [40]. Tea saponins, the core active components of CSM, are natural triterpenoid saponins that possess strong free radical scavenging capabilities and lipid oxidation inhibitory effects [41]. The present study found that an appropriate dose of fermented CSM significantly enhanced the total antioxidant capacity (T-AOC) and total superoxide dismutase (T-SOD) activities in the serum and jejunal mucosa of nursery pigs. This result is consistent with the findings of Wang et al. [42] in dairy cows (tea saponins linearly increased plasma SOD activity and decreased MDA content) and the study by Chi et al. [43] in broilers (tea saponins comprehensively enhanced the body’s antioxidant defense capabilities). More importantly, the current study further reveals the unique advantages of fermentation processing: the microbial fermentation process itself can release phenolic active compounds and antioxidant peptides by degrading plant cell walls, directly improving the body’s T-AOC and upregulating endogenous antioxidant enzyme activities [44]. Therefore, the enhancement of antioxidant functions in nursery pigs by fermented CSM is the result of synergistic effects between the inherent activity of tea saponins and fermentation products, which offers more comprehensive antioxidant advantages compared with the use of tea saponins alone or unfermented CSM. Regarding immune regulation, this study confirmed the dose-dependent immunomodulatory effects of fermented CSM. The FCSM2.5 group showed a tendency to decrease the level of the pro-inflammatory cytokine IL-1β, while simultaneously increasing the levels of the anti-inflammatory cytokine IL-10 and secretory immunoglobulin A (sIgA), as well as upregulating the expression of related genes. This highly aligns with existing studies showing that tea saponins can regulate inflammatory cytokine secretion and enhance lymphocyte proliferation [42,43], and that microbial fermentation products can promote IL-10 secretion and increase sIgA levels [45]. A novel contribution of this study is systematically revealing the molecular mechanisms by which low-dose fermented CSM promotes anti-inflammatory responses and humoral immunity while suppressing local inflammation through the synergistic activities of tea saponins and fermentation products, at both the protein and gene transcription levels. However, at a high inclusion level of 7.5%, the level of IFN-γ in the jejunal mucosa significantly increased and the IL-1β level tended to increase, whereas the levels of IL-10 and IgA in the jejunal mucosa, as well as serum IL-10 and IgG, significantly decreased. This clearly indicates that the residual ANFs at high doses have exceeded the body’s tolerance threshold, shifting from immunomodulation to immunosuppression, triggering local intestinal inflammation, disrupting immune homeostasis, and inhibiting immunoglobulin synthesis [35]. The close association between serum immunoglobulin levels and intestinal immune-related gene expression [46] was also corroborated in this study: the transcription levels of intestinal IGHA, IGHG, and IL10 genes were significantly upregulated in the FCSM2.5 group, whereas the opposite was observed in the high-dose group. This finding fills the gap in existing research regarding the regulation of immune genes in nursery pigs by fermented CSM, and deepens the understanding of its immunomodulatory mechanisms—namely, that its regulatory effects are clearly dose-dependent, and the molecular-level changes are highly consistent with phenotypic indicators.

**Figure 5:**
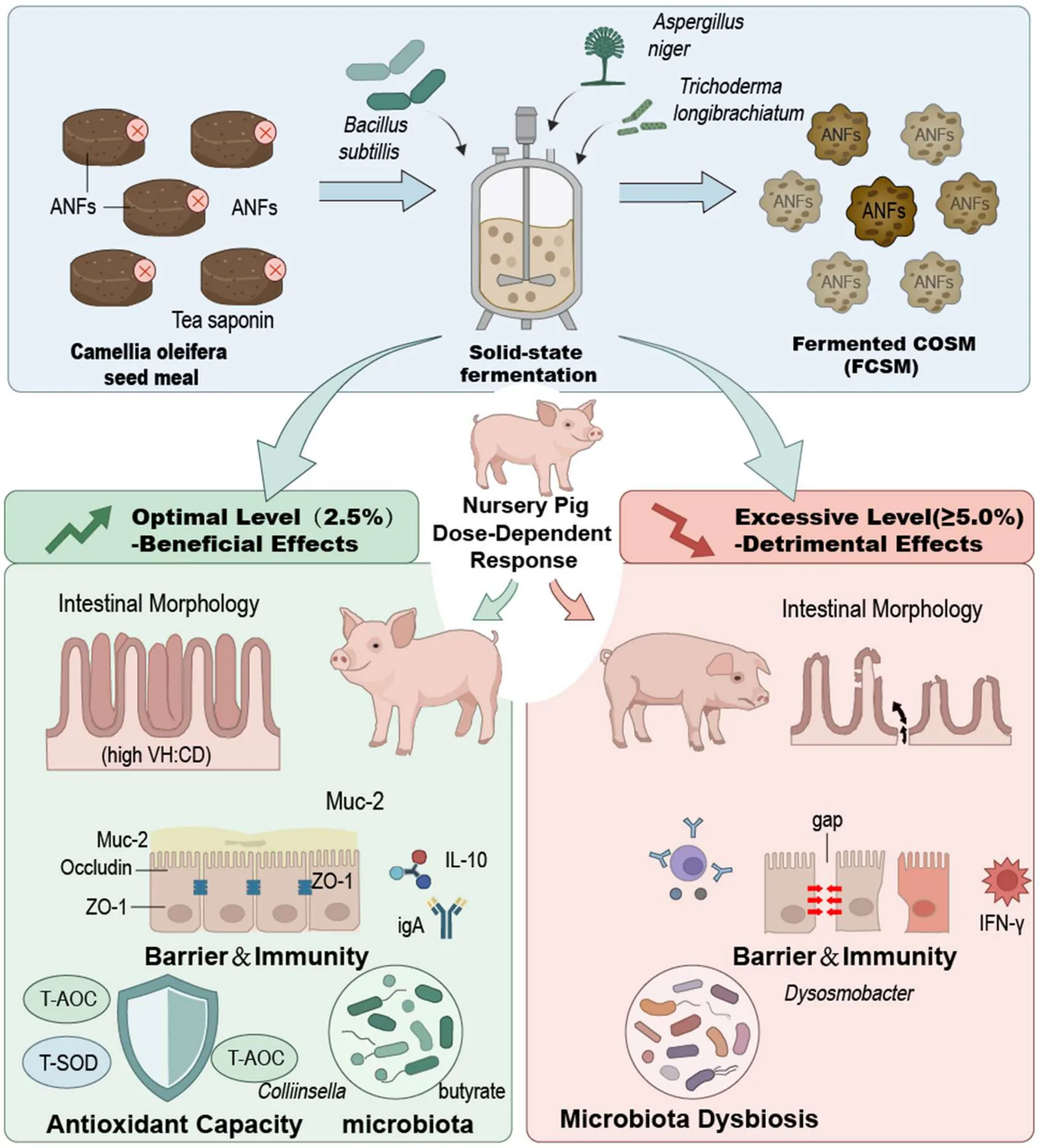
Mechanistic model of fermented *Camellia oleifera* seed meal (FCSM) regulating growth performance and intestinal health in nursery pigs. Low-dose FCSM (2.5%) reduces tea saponin, improves intestinal barrier and microbiota, and enhances growth. Excessive FCSM (≥5.0%) causes residual antinutrient-induced inflammation and growth inhibition.

A tightly coordinated interactive network exists among the intestinal microbiota, short-chain fatty acid (SCFA) metabolism, intestinal morphological development, and mucosal barrier function. The microbiota produces SCFAs through metabolism, which can regulate the development of the intestinal villus–crypt structure and barrier integrity, thereby maintaining intestinal homeostasis and healthy growth [47,48,49]. As the core segment for nutrient absorption, the morphological structure of the jejunum is particularly sensitive to dietary components [50]. In the present study, dietary supplementation with 2.5% fermented CSM (FCSM2.5) significantly improved jejunal morphology by increasing the villus height and the VH:CD ratio. This is consistent with the findings of Stanev et al. [51], who confirmed that plant saponin additives can increase the intestinal villus height in broilers; however, the current study extends this finding to nursery pigs and clearly defines its dose effect. At the molecular level, the FCSM2.5 treatment upregulated the gene expression of tight junction proteins (ZO-1, Occludin, and Claudin-1) and mucin (MUC2), whereas these expression levels significantly declined in the FCSM7.5 group. This mechanism is directly related to the degree of ANF degradation in CSM achieved through fermentation [52]. At low doses, the fermentation products not only enhance the intestinal antioxidant capacity to scavenge reactive oxygen species (ROS) within epithelial cells and reduce oxidative damage to tight junction proteins [53], but also exert anti-inflammatory effects to weaken the inhibition of barrier gene promoters by inflammatory signals. This promotes MUC2 synthesis, forming a synergistic defense mechanism coupled with the upregulation of the IGHA gene, which aligns with the findings of Niu et al. [54]. Conversely, at high doses, incompletely degraded ANFs induce oxidative stress and chronic inflammation, leading to barrier damage [49]. Regarding the intestinal microbiota, 16S rRNA sequencing in the present study revealed that the reshaping of the microbial community structure acts as a crucial mediator for the aforementioned beneficial effects. Although alpha diversity indices showed no significant differences among the groups, the decreasing trend of the Shannon index in the FCSM2.5 group was not indicative of microbiota dysbiosis, but rather correlated with the directional enrichment of the butyrate-producing genus *Collinsella*. This phenomenon supports the perspective that “the functional structure of the microbiota is more critical than mere diversity metrics” [55], challenging the simplified dogma that “higher diversity strictly equates to better health.” PCoA combined with ANOSIM showed weak statistical differences in microbial community composition among groups (R = 0.14691, *p* = 0.011). The overall intestinal microbiota maintained high similarity across all treatments, and only the relative abundance of several specific genera was altered, whereas the Venn diagram showed that core shared operational taxonomic units (OTUs) accounted for function and jejunal morphological development indices (*p* < 0.05), and significantly negatively correlated with pro-inflammatory factors and feed-to-gain ratio (*p* < 0.05). These correlation results suggested that the altered abundance of these differential genera was closely linked to the intestinal health and growth performance of piglets. Specifically, *Collinsella*, enriched in the FCSM2.5 group, is a well-recognized butyrate-producing genus that generates butyrate via carbohydrate fermentation; as the primary energy source for colonic epithelial cells, butyrate can strengthen intestinal barrier integrity and exert anti-inflammatory effects [56], which is consistent with the positive correlations between *Collinsella* abundance and intestinal barrier-related gene expression as well as villus height observed in this study, and also matches the quadratic response of colonic butyric acid content to graded FCSM supplementation. *Streptococcus*, the characteristic genus of the FCSM5 group, mainly participates in intestinal carbohydrate fermentation and lactic acid production, which helps lower intestinal pH, inhibit pathogenic bacteria colonization and indirectly promote nutrient digestion and absorption, corresponding to its positive correlations with multiple nutrient digestibility indices. *Lactobacillus*, *Weissella* and *Bacillus* were predominantly enriched in the FCSM7.5 group; although these genera are generally regarded as probiotics that can secrete digestive enzymes and regulate immune response [20], their enrichment at high inclusion levels may be a compensatory response to undigested fiber and residual anti-nutritional factors, which cannot reverse the overall negative effects induced by excessive saponin toxicity. Consistently, *Dysosmobacter* enriched in the FCSM7.5 group was extremely significantly positively correlated with pro-inflammatory factors (*p* < 0.01) and significantly negatively correlated with intestinal barrier function and growth performance (*p* < 0.05), providing direct microbiological evidence for high-dose fermented CSM-induced intestinal inflammation and growth inhibition. This confirms that the fermentation treatment achieves community reshaping by regulating the relative abundances of key genera while maintaining the homeostasis of the core microbiota [57]. Crucially, this study tightly linked microbiota alterations with host phenotypes, revealing the microbiological mechanism by which different doses of fermented CSM exert dose-dependent effects by regulating the abundance of specific genera. *Collinsella*, enriched in the FCSM2.5 group, was significantly positively correlated with intestinal barrier-related indicators. Butyric acid tended to increase at moderate FCSM inclusion levels, which may serve as the main energy source for intestinal epithelial cells and further improve intestinal barrier function [56]. Meanwhile, the significant reduction in isocaproic acid content in the low- and medium-dose groups was consistent with the decreasing trend in the abundance of *Pseudomonadota*, further confirming that fermented CSM can optimize the intestinal fermentation pattern and reduce the accumulation of protein putrefaction metabolites. In summary, as illustrated in Figure 5, this study systematically elucidates the regulatory axis of “fermented CSM–intestinal microbiota–host health.” An appropriate dose (2.5%) enriches beneficial bacteria such as *Collinsella,* optimizes SCFA metabolism, and synergistically enhances intestinal barrier and immune functions, ultimately promoting growth. Conversely, high doses lead to microbiota dysbiosis (e.g., enrichment of *Dysosmobacter*) due to the accumulation of ANFs, thereby triggering inflammation and barrier damage, which suppresses growth performance. From a practical application perspective, fermented CSM exhibits favorable economic benefits and industrial applicability; as a low-value agricultural by-product, CSM costs only 30–40% of soybean meal, and after simple solid-state fermentation treatment, 2.5% dietary inclusion can reduce feed cost by approximately 1.5–2% without compromising the growth performance of nursery pigs; in addition, the solid-state fermentation process used in this study has low requirements for production equipment, is simple to operate and is easy for large-scale promotion in commercial feed mills, indicating that fermented CSM has broad application prospects as a novel alternative protein feed ingredient. Some limitations of the current study should be noted. The absence of an unfermented CSM control means the observed effects represent the combined action of inherent CSM bioactive components and fermentation-induced improvements, and the independent contribution of fermentation remains to be further clarified. Comprehensive compositional and microbial characterization of the fermented product was not fully covered in this work, and dietary energy and amino acid levels were not completely equalized across treatments, though the opposing trends of energy level and growth performance support that residual anti-nutritional factors dominate the dose-dependent effect. Future studies with unfermented CSM controls and isoenergetic/isonitrogenous diets, combined with multi-omics strategies including metagenomics, metabolomics and transcriptomics, will help to more accurately evaluate the application value of fermented CSM and systematically elucidate its underlying mechanisms. 

## 5. Conclusions

The present study demonstrated that dietary supplementation with compound probiotic-fermented *Camellia oleifera* seed meal (CSM) exerted a dose-dependent effect on growth performance, antioxidant status, immune function, and intestinal health in nursery pigs, following an inverted U-shaped response pattern. Inclusion of 2.5% fermented CSM effectively reduced the adverse effects of anti-nutritional factors while enhancing nutrient digestibility, antioxidant capacity, and immune responses. These beneficial effects were associated with improved jejunal morphology, increased expression of barrier- and anti-inflammatory-related genes, modulation of colonic microbiota composition, enrichment of the butyrate-producing genus *Collinsella,* and improved short-chain fatty acid metabolism. In contrast, dietary inclusion levels of 5.0% or above adversely affected growth performance and intestinal health, likely due to the residual effects of anti-nutritional factors. Therefore, under the conditions of the present study, 2.5% is the optimal inclusion level for fermented CSM, and this material can be developed as a potential novel feed ingredient for nursery pig diets.

## Figures and Tables

**Table 1:** Composition and nutrient levels of the basal diets.

Items	CON ^1^	FCSM2.5 ^2^	FCSM5 ^3^	FCSM7.5 ^4^
Ingredient composition (%)				
Extruded corn	64.00	62.00	60.00	58.00
Soybean meal	19.00	18.50	18.00	17.50
Fermented soybean meal	9.00	9.00	9.00	9.00
Fish meal	4.00	4.00	4.00	4.00
FCSM	0.00	2.50	5.00	7.50
Dicalcium phosphate	1.00	1.00	1.00	1.00
Limestone	1.10	1.10	1.10	1.10
L-Lysine-HCl	0.30	0.30	0.30	0.30
DL-Methionine	0.10	0.10	0.10	0.10
L-Threonine	0.10	0.10	0.10	0.10
L-Tryptophan	0.05	0.05	0.05	0.05
Premix ^5^	1.35	1.35	1.35	1.35
Total	100.00	100.00	100.00	100.00
Calculated nutrient levels				
Digestible energy (kcal/kg)	3494.42	3520.37	3546.32	3572.27
Digestible energy (MJ/kg)	14.62	14.73	14.84	14.95
Crude protein, CP (%)	20.19	20.17	20.15	20.12
Neutral detergent fiber (NDF, %)	9.73	10.34	10.96	11.58
Acid detergent fiber (ADF, %)	3.43	3.89	4.36	4.82
Calcium (%)	0.78	0.79	0.79	0.80
Total phosphorus (%)	0.57	0.57	0.57	0.58
Available phosphorus (%)	0.42	0.42	0.41	0.41
Lysine (%)	1.39	1.39	1.39	1.39
Threonine (%)	0.84	0.84	0.84	0.83
Methionine (%)	0.45	0.44	0.45	0.44
Actual Measurement of Chemical Composition Analysis				
Saponin (mg/kg) ^6^	0	495	946	1388

^1^ CON: corn-soybean meal (SBM) basal diet with no fermented *Camellia oleifera* seed meal (FCSM). ^2^ FCSM2.5: basal diet supplemented with 2.5% FCSM, which replaced 2.0% extruded corn and 0.5% SBM. ^3^ FCSM5.0: basal diet supplemented with 5.0% FCSM, which replaced 4.0% extruded corn and 1.0% SBM. ^4^ FCSM7.5: basal diet supplemented with 7.5% FCSM, which replaced 6.0% extruded corn and 1.5% SBM. ^5^ Premix supplied the following nutrients per kilogram of diet: 8400 IU vitamin A, 1800 IU vitamin D3, 20 IU vitamin E, 2.0 mg vitamin K3, 1.6 mg vitamin B1, 4.0 mg vitamin B2, 2.0 mg vitamin B6, 20.0 μg vitamin B12, 20.0 mg niacinamide, 0.08 mg biotin, 1.0 mg folic acid, and 10.0 mg pantothenic acid. Trace minerals provided included 100 mg Fe, 6 mg Cu, 100 mg Zn, 4 mg Mn, 0.14 mg I, 0.3 mg Se, and 0.4 mg Co per kilogram of complete diet. In addition, the premix contained choline chloride, phytase, and antioxidant additives. ^6^ Saponin contents in diets are measured values of complete feed. Slight differences from theoretical calculated values are attributed to feed matrix effect and analytical recovery.

**Table 2:** Effects of Fermented *Camellia oleifera* Seed Meal on Growth Performance and Nutrient Digestibility of Nursery Pigs.

Items	Dietary Treatments				SEM	*p*-Value		
	CON	FCSM2.5	FCSM5	FCSM7.5		ANOVA	Linear	Quadratic
IBW (kg)	15.48	15.45	15.46	15.47	0.02	0.982	0.917	0.749
FBW (kg)	36.02 ^a^	35.50 ^a^	29.76 ^b^	24.67 ^c^	0.92	<0.001	<0.001	0.001
ADG (kg/d)	0.50 ^a^	0.49 ^a^	0.34 ^b^	0.22 ^c^	0.02	<0.001	<0.001	0.001
ADFI (kg/d)	1.14 ^a^	1.09 ^a^	1.00 ^b^	0.79 ^c^	0.03	<0.001	<0.001	<0.001
F/G	2.28 ^c^	2.23 ^c^	2.94 ^b^	3.61 ^a^	0.13	<0.001	<0.001	0.029
Ash (%)	29.15	25.55	29.51	31.06	0.97	0.250	0.293	0.194
CP (%)	84.70 ^ab^	87.23 ^a^	84.95 ^ab^	82.36 ^b^	0.65	0.036	0.050	0.304
EE (%)	87.25	80.32	79.87	79.32	1.80	0.549	0.194	0.420
NDF (%)	44.43 ^b^	48.18 ^a^	46.14 ^ab^	44.29 ^b^	0.47	<0.001	0.343	<0.001
ADF (%)	31.92	31.74	31.36	33.21	0.89	0.911	0.667	0.606
Ca (%)	62.71	64.64	65.57	65.14	1.07	0.809	0.456	0.628
*p* (%)	69.02	66.35	68.12	69.64	1.70	0.933	0.819	0.571

Means within a row with different lowercase letter superscripts differ significantly (*p* < 0.05), whereas those sharing a common letter are not significantly different (*p* > 0.05). CON = basal diet; FCSM2.5, FCSM5, and FCSM7.5 = basal diet supplemented with 2.5%, 5.0%, and 7.5% fermented *Camellia oleifera* seed meal, respectively. IBW = initial body weight; FBW = final body weight; ADG = average daily gain; ADFI = average daily feed intake; F/G = feed-to-gain ratio; CP = crude protein; EE = ether extract; NDF = neutral detergent fiber; ADF = acid detergent fiber; Ca = calcium; *p* = phosphorus; SEM = standard error of the mean.

**Table 3:** Effects of Fermented *Camellia oleifera* Seed Meal on the Antioxidant Capacity of Nursery Pigs.

Items	Dietary Treatments				SEM	*p*-Value		
	CON	FCSM2.5	FCSM5	FCSM7.5		ANOVA	Linear	Quadratic
**Jejunum**								
T-AOC (U/mg prot)	0.07 ^c^	0.10 ^b^	0.11 ^ab^	0.12 ^a^	0.01	0.001	<0.001	0.360
T-SOD (U/mg prot)	96.94 ^c^	140.08 ^a^	125.55 ^b^	125.40 ^b^	4.98	<0.001	<0.001	0.002
GSH-Px (U/mg prot)	100.08	100.94	111.56	111.71	2.42	0.156	0.033	0.942
CAT (U/mg prot)	27.59	26.36	27.99	23.02	2.60	0.928	0.895	0.728
MDA (nmol/mg prot)	2.07	2.01	1.91	1.84	0.09	0.821	0.365	0.983
**Serum**								
T-AOC (U/mL)	0.90 ^b^	1.06 ^a^	1.11 ^a^	1.12 ^a^	0.24	0.012	0.002	0.067
T-SOD (U/mL)	120.67 ^c^	205.37 ^a^	179.26 ^b^	170.32 ^b^	5.92	<0.001	<0.001	<0.001
GSH-Px (U/mL)	384.87	400.37	417.69	437.09	30.01	0.961	0.607	0.978
CAT (U/mL)	16.25	16.33	14.30	11.71	0.92	0.218	0.73	0.461
MDA (nmol/mL)	2.18	2.05	2.19	2.24	0.09	0.927	0.730	0.662

Within a row, means with different lowercase superscripts differ significantly (*p* < 0.05), whereas those sharing the same superscript do not differ significantly (*p* > 0.05). CON = basal diet; FCSM2.5, FCSM5, and FCSM7.5 = basal diet supplemented with 2.5%, 5.0%, and 7.5% fermented *Camellia oleifera* seed meal, respectively. T-AOC = total antioxidant capacity; T-SOD = total superoxide dismutase; GSH-Px = glutathione peroxidase; CAT = catalase; MDA = malondialdehyde; SEM = standard error of the mean. *n* = 7.

**Table 4:** Effects of fermented *Camellia oleifera* seed meal on immune factors in nursery pigs.

Items	Dietary Treatments				SEM	*p*-Value		
	CON	FCSM2.5	FCSM5	FCSM7.5		ANOVA	Linear	Quadratic
**Jejunum**								
IL-1β (pg/mg prot)	26.64	22.63	26.71	28.63	0.89	0.060	0.167	0.072
IL-6 (pg/mg prot)	135.94	131.65	130.10	129.57	4.00	0.946	0.592	0.838
IL-10 (pg/mg prot)	26.19 ^b^	34.81 ^a^	30.41 ^ab^	26.01 ^b^	1.27	0.045	0.586	0.009
TNF-α (pg/mg prot)	23.92	19.65	24.97	25.61	1.14	0.198	0.353	0.288
IFN-γ (pg/mg prot)	4.98 ^b^	4.91 ^b^	6.06 ^ab^	7.14 ^a^	0.28	0.001	<0.001	0.149
IgA (mg/g prot)	1.33 ^c^	1.87 ^a^	1.67 ^ab^	1.57 ^bc^	0.07	0.006	0.171	0.003
IgG (mg/g prot)	5.04	5.43	5.37	4.39	0.20	0.228	0.260	0.096
IgM (mg/g prot)	1.72	1.68	1.80	1.80	0.05	0.838	0.472	0.888
**Serum**								
IL-1β (pg/mL)	144.55	143.90	145.54	150.15	2.87	0.888	0.509	0.664
IL-6 (pg/mL)	735.80	727.66	687.89	665.98	15.78	0.433	0.137	0.835
IL-10 (pg/mL)	161.57 ^a^	175.61 ^a^	156.50 ^a^	129.15 ^b^	5.70	0.004	0.023	0.385
TNF-α (pg/mL)	128.00	127.59	134.29	134.82	2.82	0.775	0.363	0.942
IFN-γ (pg/mL)	42.06	40.26	40.92	43.19	1.16	0.863	0.731	0.425
IgA (g/L)	9.12 ^b^	10.93 ^a^	10.89 ^a^	10.40 ^ab^	0.27	0.018	0.90	0.021
IgG (g/L)	26.03 ^a^	26.20 ^a^	22.39 ^ab^	16.47 ^b^	1.25	0.007	0.002	0.151
IgM (g/L)	1.02	1.04	0.99	1.00	0.19	0.865	0.622	0.788

Within a row, means with different lowercase superscripts differ significantly (*p* < 0.05). CON = basal diet; FCSM2.5, FCSM5, and FCSM7.5 = basal diet supplemented with 2.5%, 5.0%, and 7.5% fermented *Camellia oleifera* seed meal, respectively. IL-1β = interleukin-1β; IL-6 = interleukin-6; IL-10 = interleukin-10; TNF-α = tumor necrosis factor-α; IFN-γ = interferon-γ; IgA = immunoglobulin A; IgG = immunoglobulin G; IgM = immunoglobulin M; SEM = standard error of the mean. *n* = 7.

**Table 5:** Effects of fermented *Camellia oleifera* seed meal on the intestinal histomorphology of nursery.

Items (µm)	Dietary Treatments				SEM	*p*-Value		
	CON	FCSM2.5	FCSM5	FCSM7.5		ANOVA	Linear	Quadratic
Duodenum								
Villus height	507.06	531.86	510.48	505.98	9.52	0.776	0.794	0.499
Crypt depth	409.55	371.66	408.91	451.98	14.60	0.335	0.217	0.176
V:C ratio	1.22	1.37	1.28	1.17	0.06	0.686	0.673	0.287
Jejunum								
Villus height	461.64 ^bc^	539.81 ^a^	520.52 ^ab^	434.96 ^c^	12.80	0.003	0.278	<0.001
Crypt depth	271.60	254.69	298.89	305.69	10.51	0.295	0.128	0.573
V:C ratio	1.62 ^b^	2.13 ^a^	1.86 ^ab^	1.53 ^b^	0.08	0.032	0.434	0.009
Ileum								
Villus height	408.77	488.44	454.56	438.96	22.97	0.705	0.829	0.370
Crypt depth	236.01	221.45	254.46	255.79	8.38	0.400	0.259	0.646
V:C ratio	1.84	2.08	1.96	1.95	0.08	0.765	0.802	0.492

Within the same row, means with different lowercase superscripts differ significantly (*p* < 0.05), whereas those sharing the same superscript are not significantly different (*p* > 0.05). CON = basal diet; FCSM2.5, FCSM5, and FCSM7.5 = basal diet supplemented with 2.5%, 5.0%, and 7.5% fermented *Camellia oleifera* seed meal, respectively. Villus height (VH); crypt depth (CD); V:C = villus height-to-crypt depth ratio; SEM = standard error of the mean. *n* = 7.

**Table 6:** Effects of Fermented *Camellia oleifera* Seed Meal on Short-Chain Fatty Acids in Nursery Pigs.

Items (μg/mg)	Dietary Treatments				SEM	*p*-Value		
	CON	FCSM2.5	FCSM5	FCSM7.5		ANOVA	Linear	Quadratic
Acetic acid	5.82	5.52	5.46	6.10	0.14	0.334	0.522	0.107
Propionic acid	2.26	2.08	2.16	2.15	0.08	0.892	0.755	0.603
Butyric acid	0.84	1.02	1.05	0.84	0.04	0.128	0.919	0.025
Valeric acid	0.21	0.19	0.24	0.22	0.01	0.318	0.370	0.171
Caproic acid	0.0023	0.0023	0.0025	0.0022	0.0001	0.665	0.770	0.329
Isobutyric acid	0.14	0.13	0.15	0.13	0.01	0.175	0.488	0.241
Isovaleric acid	0.13	0.11	0.13	0.12	0.01	0.529	0.521	0.721
Isocaproic acid	0.0096 ^a^	0.0063 ^bc^	0.0061 ^c^	0.0090 ^ab^	0.0005	0.006	0.002	0.957

Within the same row, means with different lowercase superscripts differ significantly (*p* < 0.05), whereas those sharing the same superscript are not significantly different (*p* > 0.05). CON = basal diet; FCSM2.5, FCSM5, and FCSM7.5 = basal diet supplemented with 2.5%, 5.0%, and 7.5% fermented *Camellia oleifera* seed meal, respectively. SEM = standard error of the mean. *n* = 7.

## Data Availability

The raw sequencing data of colonic microbiota were deposited in the National Center for Biotechnology Information (NCBI) with the accession number PRJNA1482787.

## Supplementary Materials

The following supporting information can be downloaded at: https://www.mdpi.com/article/10.3390/ani16213432/s1. Figure S1. Schematic workflow for solid-state fermentation of Camellia oleifera seed meal, including strain activation, substrate preparation, fermentation parameters, post-treatment, and the measured tea saponin degradation efficiency before and after fermentation. Table S1. Primer sequences used for RT-qPCR detection of jejunal related genes.
