# Supplementary material for: Effects of Varying Levels of Fermented Camellia oleifera Seed Meal on Growth Performance, Biochemical Indices, and Intestinal Health in Nursery Pigs
Source: Animals (Basel). 2026 Oct 30;16(21):3432. doi: 10.3390/ani16213432 (PMC13762251; doi:10.3390/ani16213432)
Supplement: Supplementary file 1 [file animals-16-03432-s001.zip › animals-4456385-supplementary.pdf]

Supplementary Materials for “Effects of varying levels of fermented Camellia oleifera seed meal on growth performance, biochemical indices, and intestinal health in nursery pigs”

(Manuscript ID: animals-4456385)

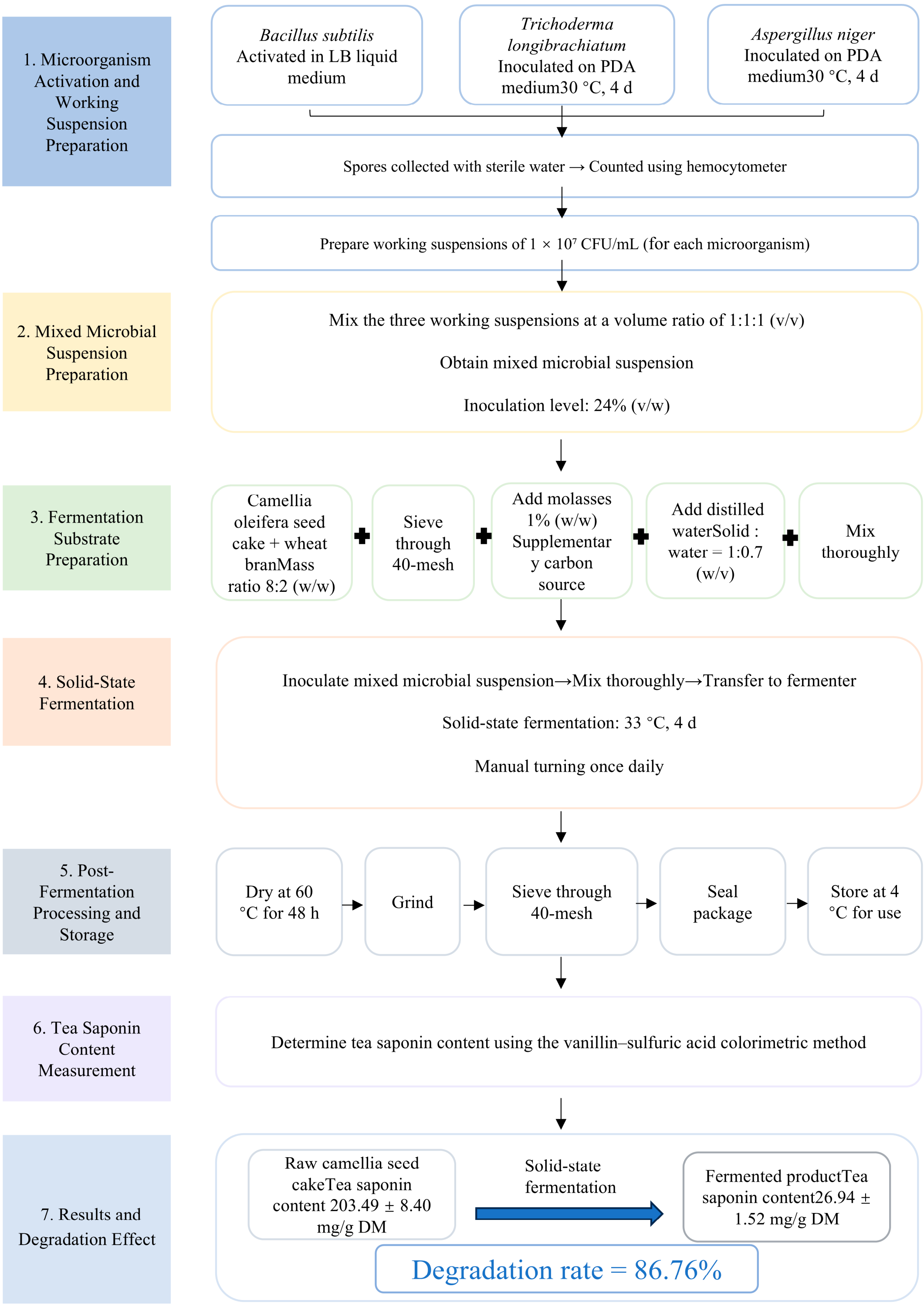

Figure S1. Schematic workflow for solid-state fermentation of Camellia oleifera seed meal, including strain activation, substrate preparation, fermentation parameters, post-treatment, and the measured tea saponin degradation efficiency before and after fermentation.

Table S1. Primer sequences used for RT-qPCR detection of jejunal related genes

Genes	Primer sequences (5' to 3')	Size, bp	Accession number
β-Actin	F: CCAGCACGATGAAGATCAAGA<br>R:AATGCAACTAACAGTCCGCCTA	178	ON164673.1
IL1β	F: CACACATGCTGAAGGCTCTC<br>R: GGGTGGGCGTGTTATCTTTC	171	NM_214055.1
IL10	F: CTGAGAACAGCTGCATCCAC<br>R: AAAGTCCTCCAGCAGAGACC	146	NM_214041.1
IL17A	F: CTCGGGCTGTATCAATGCTG<br>R: GAAATATGGCGGACGATGGG	177	NM_001005729.1
TNF-α	F: AAGGTCAACCTCCTCTCTGC<br>R: CCTCCCAGGTAGATGGGTTC	98	JF831365.1
IGHA	F: GACAAGTACCTCGTCTGGGA<br>R: GAGAAGGTGTCTCCCTGCTT	116	GU553018.1
IGHG	F: ACACCTGCAATGTCAACCAC<br>R: GGTCTTTGTTCCAACACGCT	65	AF062384.1
ZO-1	F: GGAGAGAAGTGCCAGTAGGG<br>R: TATGGCTGGCCAATCGAAGA	148	XM_021098827.1
Occludin	F: CATTATGCACCCAGCAACGA<br>R: GCACATCACGATAACGAGCA	168	NM_001163647.2
Claudin-1	F: AATTCCACCACCAACATGCC<br>R: TCAGACCACTCAGTGTGCAT	145	NM_001244539.1
MUC1	F: CTGATGGAAGTGCTGTGAGC<br>R: CTGGTGGTTGGAGAGGACAT	176	NM_001435127.1
MUC2	F: CTTGCTCTCGTGTGGAACAG<br>R: TCCTCGGGCTTGTTGATCTT	185	XM_021082584.1
MUC4	F: GAGACCACCAGAGAAACCCA<br>R: TGATGTTGTTGCAGGTGTGG	174	XM_021068272.1

Notes: F = forward primer (5′→3′); R = reverse primer (5′→3′). bp = base pair. All target gene expression levels were normalized to the reference gene *β-Actin*. NCBI GenBank accession numbers of each gene are listed accordingly.
